# Effectiveness of Acceptance and Commitment Therapy (ACT) in Mitigating Academic Procrastination and its Moderating Factors among nursing students

**DOI:** 10.17533/udea.iee.v43n2e03

**Published:** 2025-07-19

**Authors:** Manu Kohli, Navita Gupta, Gaurav Kohli, Prabhjot Saini, Kanika Guleria

**Affiliations:** 1 RN. Research Scholar. Associate Professor, Centre for Evidence-Based Practice in Healthcare. Email: manu.kohli@chitkara.edu.in. Corresponding author. https://orcid.org/0000-0002-4003-5879 Chitkara University Centre for Evidence-Based Practice in Healthcare India manu.kohli@chitkara.edu.in; 2 Ph.D. Associate Professor. Department of Allied Health Sciences. Email: navita.gupta@chitkara.edu.in. https://orcid.org/0000-0002-4163-6406 Chitkara University Department of Allied Health Sciences India navita.gupta@chitkara.edu.in; 3 RN, M.Sc. Professor, Centre for Evidence-Based Practice in Healthcare. Email: gaurav.kohli@chitkara.edu.in. https://orcid.org/0000-0003-1433-5111 Chitkara University Centre for Evidence-Based Practice in Healthcare India gaurav.kohli@chitkara.edu.in; 4 RN, Ph.D. Professor. Email: psainidmc@gmail.com. https://orcid.org/0009-0006-2494-2467 Shaheed Kartar Singh Sarabha College of Nursing India psainidmc@gmail.com; 5 RN, M.Sc. Assistant Professor, Centre for Evidence-Based Practice in Healthcare. Email: kanika.guleria@chitkara.edu.in. https://orcid.org/0000-0003-2052-8497 Chitkara University Centre for Evidence-Based Practice in Healthcare India kanika.guleria@chitkara.edu.in; 6 Chitkara School of Health Sciences, Chitkara University, Punjab, India Chitkara University Chitkara School of Health Sciences Chitkara University Punjab India; 7 Shaheed Kartar Singh Sarabha College of Nursing, Sarabha, Ludhiana, India Shaheed Kartar Singh Sarabha College of Nursing Shaheed Kartar Singh Sarabha College of Nursing Sarabha Ludhiana India

**Keywords:** procrastination, Acceptance and Commitment Therapy, self-concept, students, nursing., procrastinación, terapia de aceptación y compromiso, autoimagen, estudiantes de enfermería., procrastinação, terapia de aceitação e compromisso, autoimagem, estudantes de enfermagem.

## Abstract

**Objective.:**

To assess the effect of Acceptance and Commitment Therapy (ACT) on academic procrastination and its moderating factors among nursing students.

**Methods:**

. True experimental pre-test and post-test research design was adopted. The setting comprised of private nursing colleges of Punjab, India. A screening was done among 209 nursing students, 43 (20.57%) were procrastinators, which were assigned randomly to the intervention (*n*=19) and control (*n*=24) groups. The intervention was administered during six weeks, consisting of weekly one-hour modules that focused on the core components of ACT (Present Moment Contact, Defusion, Acceptance, Self as Context, Values, and: Commitment to Action). The effect of the intervention was measured after 6^th^ week through the scales: *(i)* Procrastination assessment Scale**,** (ii) Acceptance and action questionnaire-II (AAQ-II) and (iii) Student time management scale (STMS). A session of ACT was scheduled for the control group following the post-test. Study has been registered under Clinical Trail Registry of India (REF/2022/12/061719). Data was analysed using descriptive and inferential statistics.

**Results.:**

Post intervention assessment after 6 weeks revealed reduction in academic procrastination among the nursing students (mean difference -8.61, Standard error 0.35, *p*-value=0.01). Intervention also led to improvement in time management skills (mean difference 9.98, Standard error 0.32, *p*-value=0.001). Additionally, intervention results in improving psychological flexibility among the nursing students (mean difference -4.72, Standard error 0.49, *p*-value=0.02).

**Conclusion:**

. The study found ACT can reduce academic procrastination, and in the clinical setting, can and improve the time management, psychological flexibility among nursing students.

## Resumen

**Objetivo:**

. Evaluar el efecto de la Terapia de Aceptación y Compromiso (TAC) sobre la procrastinación académica y sus factores moderadores entre estudiantes de enfermería.

**Métodos.:**

Se realizó una investigación experimental de pre-prueba y pos-prueba. El escenario fueron las escuelas de enfermería privadas de Punjab, India. Se realizó un cribado entre 209 estudiantes de enfermería, 43 (20.57%) eran procrastinadores, que fueron asignados aleatoriamente a los grupos de intervención (*n*=19) y control (*n*=24). La intervención se administró durante seis semanas, consistiendo en módulos semanales de una hora que se centraban en los componentes básicos de CAT (Contacto con el momento presente, Defusión, Aceptación, El yo como contexto, Valores y: Compromiso para la acción). El efecto de la intervención se midió después de la 6ª semana a través de tres escalas diferentes. Se programó una sesión de ACT para el grupo de control tras la prueba posterior. El estudio se ha registrado en el Clinical Trail Registry de la India (REF/2022/12/061719). Los datos se analizaron mediante estadística descriptiva e inferencial.

**Resultados:**

. La evaluación posterior a la intervención después de 6 semanas reveló una reducción de la procrastinación académica entre los estudiantes de enfermería (diferencia media -8,61, error estándar 0,35, valor p=0,01). La intervención también mejoró las habilidades de gestión del tiempo (diferencia media: 9,98; error estándar: 0,32; valor de p: 0,001). Además, la intervención mejoró la flexibilidad psicológica de los estudiantes de enfermería (diferencia media: -4,72; error estándar: 0,49; p-valor = 0.01).

**Conclusión.:**

El estudio mostró que el ACT puede reducir la procrastinación académica y, en el entorno clínico, puede mejorar el rendimiento y el bienestar de los estudiantes en los estudiantes de enfermería.

## Resumo

**Objetivo.:**

Avaliar o efeito da Terapia de Aceitação e Compromisso (ACT) na procrastinação acadêmica e seus fatores moderadores entre estudantes de enfermagem.

**Métodos.:**

Um estudo experimental pré-teste / pós-teste foi conduzido. O cenário foram faculdades de enfermagem privadas em Punjab, Índia. Um total de 209 estudantes de enfermagem foram selecionados; 43 (20.57%) eram procrastinadores e foram aleatoriamente designados para os grupos de intervenção (*n*= 19) e controle (*n= 24).* A intervenção foi administrada por seis semanas, consistindo em módulos semanais de uma hora com foco nos componentes principais da ACT (Contato com o Momento Presente, Desfusão Cognitiva, Aceitação, Self como Contexto, Valores e Compromisso com a Ação). O efeito da intervenção foi medido após a 6ª semana por meio das escalas: (i) Escala de avaliação de procrastinação, (ii) Aceitação e ação questionário -II (AAQ-II) e (iii) Gestão do tempo do aluno escala (STMS). Uma sessão de TC foi agendada para o grupo controle após o pós-teste. O estudo foi registrado no Clinical Trilhas da Índia (REF/2022/12/061719).

**Resultados.:**

A avaliação pós-intervenção após 6 semanas revelou uma redução na procrastinação acadêmica entre estudantes de enfermagem (diferença média -8.61, erro padrão 0.35, *p*=0.01). A intervenção também melhorou as habilidades de gerenciamento de tempo (diferença média: 9.98; erro padrão: 0.32; valor de *p*<0.001). Além disso, a intervenção melhorou a flexibilidade psicológica dos estudantes de enfermagem (diferença média: -4.72; erro padrão: 0.49; *p*= 0.01).

**Conclusão:**

O estudo demonstrou que o ACT pode reduzir a procrastinação académica e, no contexto clínico, pode melhorar o desempenho e o bem-estar dos estudantes de enfermagem.

## Introduction

Procrastination refers to the individuals’ behaviour of delaying or deferring tasks until the last moment or beyond established deadlines.^1^ Academic procrastination is a procrastination related to learning. It's a tendency to postpone the completion of academic tasks without any personal justification.^2^ It is a complex phenomenon that is dominant among the learners leading to delay in their educational activities. Academic procrastination is common among the students. Over 70% of students consistently struggle with procrastination and over half of them find it difficult to stop.^3^ Some students procrastinate in most life situations while others procrastinate in specific situations. In China, nearly 40% of students are troubled by academic procrastination.^4^ Procrastination is a biggest challenge in the college students of India, affecting 28.85% of student population. The habit of delaying tasks and assignments may stop or slow down the academic progress and productivity in the students.^5^

Academic procrastination can be caused by a variety of factors, such as personality traits, poor time management skills, unpleasant emotions, contextual factors like the nature of the task or the teacher, and a clinical perspective linking the behavior to personality disorders, anxiety, and depression. Most influencing factors associated with procrastination among students are task aversion, fear of failure and perfectionism.^6^ Student procrastination in academics among nursing students can be influenced by a multitude of factors, including student characteristics, educator dynamics, assignment characteristics, institutional factors, and professional obligations. Extended classes/lectures and big clinical workload can be trigger of procrastination tendency. This reflective tendency of procrastination develops due to difficulty faced by students to manage the stress and pressure in real time. Atychiphobia and laziness are the frequent reasons for procrastination among female students while among male students it is rebellion against control.^7^

Academic procrastination exerts detrimental effects on students, resulting in underachievement, heightened psychological stress, diminished subjective well-being, negative emotional states such as anxiety, depression, and disappointment. Moreover, it can also impair physical health over time.^8^ Considering the increased occurrence of mental health challenges among college undergraduates, moderating variables such stress, overwork, and procrastination must be addressed and minimised.^9^^,^^10^ Research shows that the students who procrastinate have higher levels of anxiety. This impact both students’ mental health and academic success.^11^

There are few interventions to reduce procrastination in the college students like emotional management, cognitive Behavioural therapy, didactical interventions, etc. Past researches has found support for reducing procrastination using conventional cognitive behaviour approach among the students.^12^ A novel approach to cognitive behavioural therapy called ACT has demonstrated encouraging results in lowering academic deficits. ACT constitutes six processes of adaptation like acceptance, defusion, self as a context, psychological flexibility, values and committed actions. It is based on the psychological flexibility that helps an individual to confront the present situation and change his actions to fulfil the desired actions. Studies have reported that ACT is effective in reducing the cognitive dissonance, anxiety and depression.^13^ Studies indicate that ACT effectively reduces the inclination to procrastinate on academic task and enhances psychological flexibility. Moreover, ACT had better long-term effect in reducing academic procrastination among students than other interventions. A few ACT based interventions has shown effectiveness for academic procrastination but literature so far is minimal. 

The study examined the feasibility and impact of ACT on academic procrastination, time management, and psychological adaptability within the nursing student population. Furthermore, study also tests the correlation between academic procrastination, time management and psychological flexibility. The study hypothesized that a) there is a significant difference between post-academic procrastination scores of students in intervention group and control group. b) There is a significant relationship between academic procrastination, time management, psychological flexibility among students in intervention and control group.

## Methods

Research design and setting. An experimental approach and true experimental research (pre-test/post-test) design was opted to achieve the objectives of study. The selected settings of study were private nursing colleges of Punjab, India. These settings were at appropriate distance to avoid the contamination of subjects. Both settings were co-educational institutions and the medium of teaching was English. The settings were affiliated to Punjab Nurses Registration Council and Indian Nursing council. 

Participants and Sampling. The initial screening was carried out on 209 nursing students from two nursing colleges of, Punjab, India. There was total 43 students who met the cut off score of procrastination scale (≥48). These students were divided into clusters on the basis of their college and each cluster was randomly allocated to control and intervention groups. There were 19 students in an intervention cluster and 24 were in control group cluster. 

Instruments. Feasibility and acceptability of the study was assessed using the feasibility of Intervention measure (FIM) and acceptability of intervention measure (AIM). In the investigation following tools were used to collect the data from the participants: (i) *Socio-demographic questionnaire-* It comprised of information like age, gender, program, year of study, residential background and birth order of the students; (ii) *Procrastination assessment Scale- students (Solomn and Rothblum)* was used to measure the academic procrastination among nursing students.^14^ This scale has 2 components, *Frequency of Academic Procrastination* (*n*=18) evaluates the frequency of academic procrastination -examination preparation, term paper writing, reading assignments compliance, academic administration responsibilities, attendance, and activities in general-; and second component focuses on *its reasons* (*n*=26) -aversion to the task, lack of time management, lack of personal initiative and lack of sincerity-. Frequency of academic procrastination was measured in terms of six domains and items were rated on a 5-point scale, ranging from strongly agree [5] to strongly disagree [1]. The factors contributing to students procrastination outlines on a scale ranging from 19-44 items. These reasons were rated on 5 point rating scale with a higher the score indicating a greater tendency towards a particular reason for procrastination. The internal consistency of the scale is 0.82(Cronbach alpha).(iii) *Acceptance and action questionnaire-II (AAQ-II):* measures students’ psychological flexibility ^15^. The scale consists of 7-items to be rated on a 7-point rating scales varying from never true (1) to always true (2). Higher the score, more is the psychological inflexibility and lesser score means more psychological flexibility. The reliability coefficient of the AAQ-II is 0.957 and it has strong construct and predictive validity. (iv) *Student time management scale (STMS):*^16^ measures the time management skills of the students. It has 28 items to be rated on 6-point scale ranging from strongly agree [1] to strongly disagree [6]. The scale demonstrates a Cronbach alpha’s alpha reliability of 0.78. 

Intervention. ACT Intervention was subdivided into 6 modules. Each module was administered weekly and the duration of each session was one hour. The small groups were organised as 8-10 participants for the therapy (ACT). All the modules had a common goal i.e. to minimize the academic procrastination. These modules consisted of six core processes of the ACT. The sequence of the modules was fixed and the modules were supported by visual materials (diagrams, cartoons, etc), metaphors, homework and use of Socratic questioning. (Table 1)

**Table 1:** Details of the sessions on Acceptance and Commitment Therapy

Week	Module	Objective	Activities
1^st^ week	Module 1: Contacting the present moment	Participants will develop the conscious awareness of the present moment and will be able to perceive what is happening due to their procrastinatory behaviour.	-Dropping the anchor -Mindful eating
2^nd^ week	Module 2: Defusion	Participants will decrease their attachment from inner self and use defusion to manage the procrastinatory behaviour.	-Hands as thoughts -Leaves on a stream
3^rd^ week	Module 3: Acceptance	Participants will be be able to make room for their procrastinatory thoughts and feelings instead of running away from them.	-Passengers on a Bus, -Turn off the Struggle switch
4^th^ week	Module 4: Self as context	Participants will be able to connect with their sense of self.	-Thinking self & observing self- -The sky and the weather -There go your thoughts
5^th^ week	Module 5- Values	Participants will be able to understand the important of educational values for a meaningful life.	-Working with values -Visualizing values Imagine your eightieth birthday
6^th^ week	Module 6: Committed Action	Participants will be able to understand and make actions and set goals that really matters to them with personal values.	-Setting goals -Reason giving

Reliability of the research instruments. The reliability of the instruments was checked by Cronbach’s alpha (internal consistency). The reliability of the tools was 0.82, 0.78 and 0.957 respectively for Procrastination assessment scale for students, Student time management scale, and Acceptance & action questionnaire-II.

**Fig 1:**
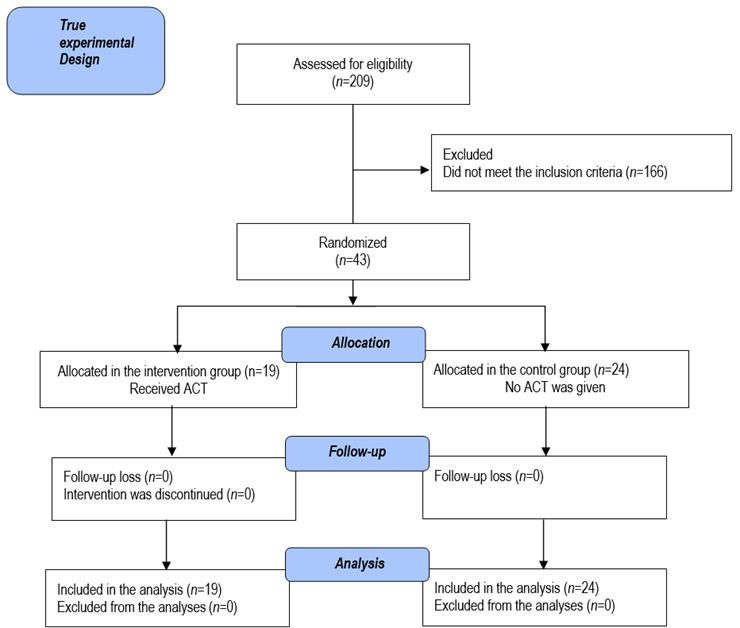
CONSORT DIAGRAM

Procedure. The data was collected in two phases following the permissions obtained from the principals of selected nursing colleges of Punjab, India. In Phase I, the student list was maintained with the assistance of class coordinators. The purpose of this investigation was explained to the concerned participants and written consent was obtained. A google form -PASS questionnaire was communicated via email to the selected students to record their responses on procrastination. Students were classified into procrastinators (≥48) or non-procrastinators (<48) on the basis of cut-off scores. In the phase II, socio demographic sheet, time management scale and the acceptance and action questionnaire II were given to selected students for baseline assessment. The selected colleges were randomly allocated in control and intervention groups as clusters. The structured intervention, ACT was given to the intervention group for six weeks, consisting of weekly one hour module. These modules (Table 2) focus on six core components of ACT and a worksheet for practicing the specific skills i.e. visual material, metaphors, homework, and Socratic questionnaire. No intervention was given to the control group until the post-test and ACT sessions were planned for the students in control group after the post-test. The study was conducted using the guidelines outlined in the CONSORT (Consolidated Standards of Reporting Trials) statement. (Figure 1)

Statistical Analysis. Analysis of the data was done using the statistical analysis software SPSS 24 version. Data was reviewed for outliers and missing values. For descriptive statistics- mean, standard deviation and percentage was used. Normal distribution of data was checked by using Kolmogorov-Smirnov test and data was distributed normally(p>0.05). For inferential statistics, adjusted Chi-square test was applied to check the homogeneity between the groups. The differences between the groups were measured by paired t-test, independent t-test and ANOVA. 

Ethics. The study received approval from Instituition Ethics committe of Chitkara University, Punjab (IHEC/DHR/CU/PB/22/112 dated 16th November, 2022). Permissions were taken from the selected colleges of nursing and informed consent was taken from the participants involved in the study. Confidentiality of the information was maintained. Study has been registered under Clinical Trail Registry of India (REF/2022/12/061719 dated 25^th^ April, 2023).

## Results

Screening. The screening was done among 209 nursing students, majority of the students 166 (79.43%) were non procrastinators and one-fourth 43 (20.57%) were procrastinators. Study results revealed that 24.88%, 38.2%, 29.19%, 39.22%, 28.22% and 20.57% of the students always procrastinate in the six domains of procrastination (i.e. studying for exams, writing term papers, keeping up with weekly reading assignments, academic administrative tasks, attendance tasks, school activities in general respectively). 

Demographic characteristics. To check the homogeneity between the two groups, adjusted chi-square test was used and no difference was found between the groups. All the variables were matched i.e. age (*p=*0.077), gender (*p=* 0.05*9*), year of study (*p=*0.279), residential background (*p=*0.652), birth order (*p=0*.346). (Table 2)

**Table 2:** Demographic characteristics of students in Intervention and control groups

Variables	Intervention Group *n* (%)	Control group *n* (%)	df	(^2^	p Value
Age (in years) 18-21 22-25	14 (73.7) 05 (26.3)	7 (29.2) 17(70.8)	1	3.132	0.077
Gender Male Female Others	08 (42.1) 11(57.9) 0	7 (29.2) 17 (70.8) 0	1	0.277	0.599
Program Name B.Sc. Nursing (Basic) B.Sc. Nursing (Post Basic)	19 (100) 0	24 (100) 0	-	-	-
Year of Study 1^st^ year/semester 2^nd^ semester 3^rd^ semester 2^nd^ year 3^rd^ year 4^th^ year	1 (5.26) 0 4 (21.05) 0 10 (52.64) 4 (21.05)	4 (16.8) 1 (4.1) 1 (4.1) 6 (25) 0 12 (50)	5	14.356	0.279
Residential background Hostel Home P.G Rented accommodation	8 (42.1) 9 (47.37) 0 2 (10.53)	8 (33.3) 15 (62.5) 0 1 (16.8)	2	0.854	0.652
Birth Order 1st 2nd 3^rd^ 4^th^ & more	11 (57.9) 8 (42.1) 0 0	11 (45.8) 11 (45.8) 0 2 (8.4)	2	2.122	0.346

Reasons of Academic procrastination. Aversiveness of the task and lack of personal initiative had positive effect on academic procrastination. For each 1% increase in aversiveness of task there is an increase of 4.37% of academic procrastination and for every 1% increase in lack of personal initiatives there is increase of 2.84 % of academic procrastination. (Table 3)

**Table 3:** Multiple linear regression analysis between academic procrastination and reasons of procrastination

Variables	Unstandardized Coefficient		Standardized coefficient	t- stat	p- value
	B	Standard error	Beta		
Academic procrastination (Constant)	1.584	0.85		1.21	0.275
Aversiveness of the task	0.437	0.42	0.75	4.76	0.001
Poor time management	0.249	0.63	0.37	1.39	0.241
Lack of Sincerity	0.552	0.57	0.53	0.78	0.182
Lack of personal Initiative	0.284	0.49	0.87	5.46	<0.001
Dependent variable: academic procrastination. Predictors: Aversiveness of task, poor time management, lack of sincerity and lack of personal initiatives

Acceptability and feasibility of intervention. In acceptability of intervention measure, 78% of the nursing students agree that the ACT intervention meets my approval and 83 % of nursing students completely agree that ACT intervention was appealing to them. In feasibility of intervention measure, 84.1% of nursing students completely agree that ACT intervention is a valuable measure and 86.4 % of them would recommend this to their friends dealing with academic procrastination

Effectiveness of Acceptance and commitment therapy. Post intervention assessment after 6 weeks revealed that ACT intervention effectively reduced academic procrastination among the nursing students, while also enhancing their time management skills and psychological flexibility.(Table 4) 

**Table 4:** Post-test scores of academic procrastination, time management and psychological flexibility in the Intervention and Control groups

Variables	Groups	Mean Score	S. D	Mean difference	Standard error	df	t-value	***p*-value**
Academic procrastination	Intervention	38.68	2.750	-8.61	0.35	41	11.903	0.01
	Control	47.29	1.916					
Time management	Intervention	107.69	9.093	9.98	0.32	41	8.548	0.001
	Control	97.71	7.215					
Psychological flexibility	Intervention	16.21	3.823	-4.72	0.49	41	6.179	0.02
Control	20.93	4.620

Correlation Academic procrastination, psychological flexibility, time management. Results depicted significant moderate positive correlation of academic procrastination with psychological flexibility (r=0.519, *p*=0.02) i.e. higher the academic procrastination score, lower is the psychological. There was significantly negative relationship between academic procrastination and time management. (r=-0.652, *p*<0.04) i.e. higher the academic procrastination, lower was the time management score. No statistically significant association was found between psychological flexibility and time management (r=0.212, *p*=0.3845)

*Association of academic procrastination, time management and psychological flexibility with demographic variables*

Association of academic procrastination, time management and psychological flexibility scores with selected variables of the nursing students (i.e. age (in year), gender, program name, year of study, residential background, and birth order), was calculated using t-test/F-test. There was significant association of the pretest academic procrastination score and residential background in the intervention group. Academic procrastination was more in the students living in the hostel. There was no significant association of academic procrastination scores with their other demographic variables in intervention and control group.

There was no association of time management and psychological flexibility scores with the selected demographic variables in intervention and control group.

## Discussion

The primary findings of study described the prevalence of procrastination among nursing students. This current study assessed that the prevalence of procrastination was 20.57% in nursing students. These findings exhibit a high prevalence of academic procrastination in literature among nursing students, with 45.72% experiencing moderate to severe procrastination.^17^ 29.25% in medical students had academic procrastination.^18^ The difference can be due to the use of different research instruments. The most common reason for academic procrastination among the students was task aversion. These findings align with the study conducted in 2022 in Iran that task aversion is the reason of procrastination.^19^

Another major aspect explored through the study was to find the effect of ACT on academic procrastination. Study results concluded that ACT demonstrated efficacy in reducing academic procrastination among nursing students. The results concurs with research studies appraising the efficacy of ACT and found medium effect size for procrastination among college students in India and elsewhere.^20^ ACT leads students towards values and worthwhile life objectives by teaching them to recognize and identify their judgements and to present them as the internal mind and world and their defusion and lack of fusion with them.

The results indicated that the ACT intervention led to an improvement in the psychological flexibility scores among the nursing students. These findings are in line with the studies that showed the ACT intervention can improve wellbeing^21^ and psychological flexibility.^22^ Findings suggested that Acceptance and commitment therapy may promote the wellbeing, behaviour modification and psychological flexibility of the nursing students. Values are one of the main core components of ACT, and values serve as the cornerstone for developing psychological flexibility. ACT can assist the students in becoming aware of their values and recognizing the important things in their lives.

Academic procrastination results from interaction of multiple components like behavioral, cognitive and affective. Many factors are associated academic procrastination. A negative relationship found between academic procrastination and time management. ^23^ The results align with the present study. Students who procrastinate their academic tasks tend to have lower time management skills. The relationship between academic procrastination and psychological flexibility aligns with the findings, psychological inflexibility may lead to academic procrastination leads among the students.^24^ Therefore ACT practitioners focus on psychological flexibility when working with students impacted by procrastination.

The study reported association of residential background with pretest academic procrastination scores in the intervention group. None of the other demographic variables exhibited association with pretest academic procrastination scores in either group. Indeed, the results are consistent with a study in which academic procrastination happen irrespective of gender and it is more common among the students living in the hostel.^25^ The strength of study is that ACT intervention modules and the tools used for data collection were standardized and validated before the use for Indian students from the clinical psychiatrist practicing ACT. 

Conclusion. The study highlights the significant impact of ACT on academic procrastination, time management, and psychological flexibility among nursing students. ACTs strong treatment of academic problems through acceptance of difficult cognitions and valuing action can be infused into nursing curriculum, support services and staff development for enhancing student performance and well-being. The application of ACT within the clinical setting is able to assist nursing students to manage stress and prevent burnout. 

Financial support: No any financial support/funding was received for this study.
